# Supplementary material for: Spermidine and Rapamycin Reveal Distinct Autophagy Flux Response and Cargo Receptor Clearance Profile
Source: Cells. 2021 Jan 7;10(1):95. doi: 10.3390/cells10010095 (PMC7827520; doi:10.3390/cells10010095)
Supplement: Supplementary file 1 [file cells-10-00095-s001.pdf]

Supplementary figures:

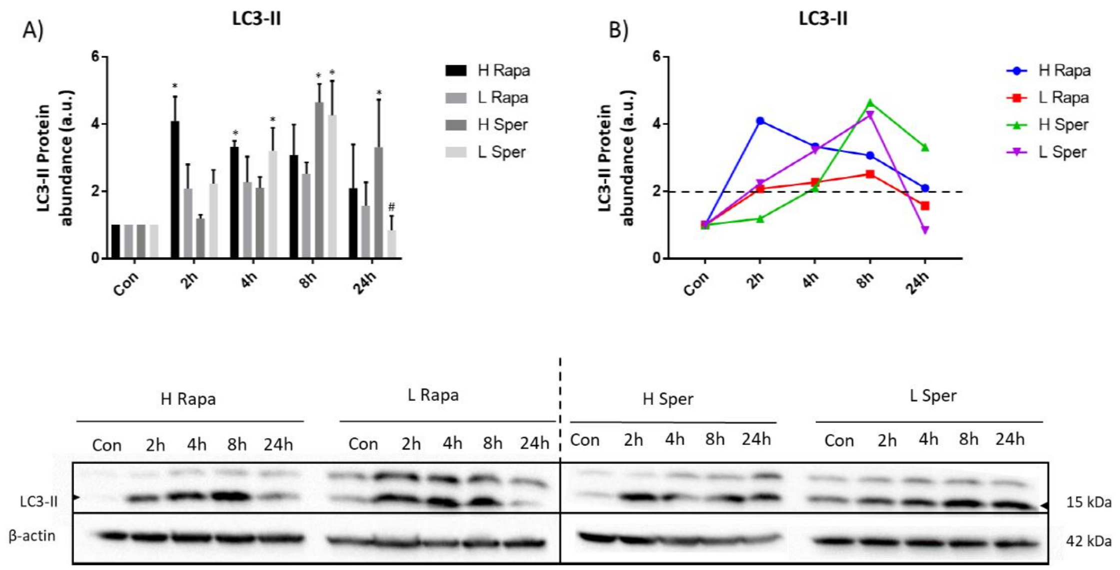

Figure S1: Western blot analyses of LC3-II abundance levels over 24 hours: Representative immunoblots are shown, indicating the relative abundance levels of LC3-II at 2, 4, 8 and 24 hours post-treatment using 1 µM Rapamycin (H Rapa), 10 nM Rapamycin (L Rapa), 20 µM Spermidine (H Sper) and 5 µM Spermidine (L Sper). **A)** Bar graphs and **B)** Line graphs are used to represent the data over time. N=3, * p<0.05 vs Con, # p<0.05 vs H Sper 24h.

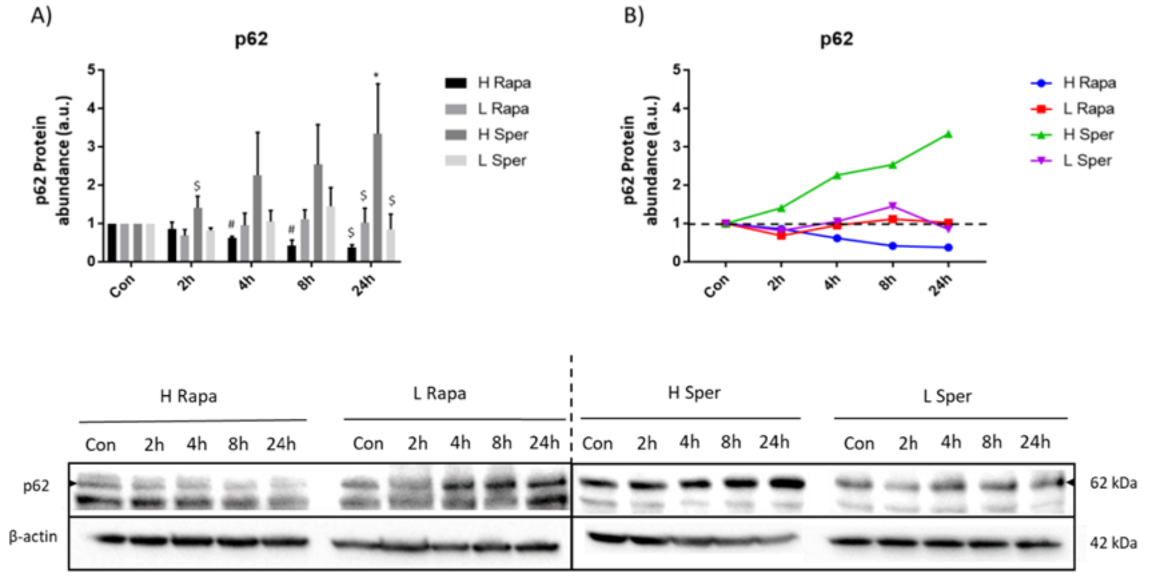

Figure S2: Western blot analysis of relative p62 abundance levels over 24 hours. Representative immunoblots are shown, indicating the relative abundance levels of p62 at 2, 4, 8 and 24 hours post-treatment using 1 µM Rapamycin (H Rapa), 10 nM Rapamycin (L Rapa), 20 µM Spermidine (H Sper) and 5 µM Spermidine (L Sper). **A)** Bar graphs and **B)** Line graphs are used to represent the data over time. N=3, *p<0.05 vs Con, #p<0.05 vs corresponding H Sper group at the same time points. $p<0.05 vs H Sper 24 hours.

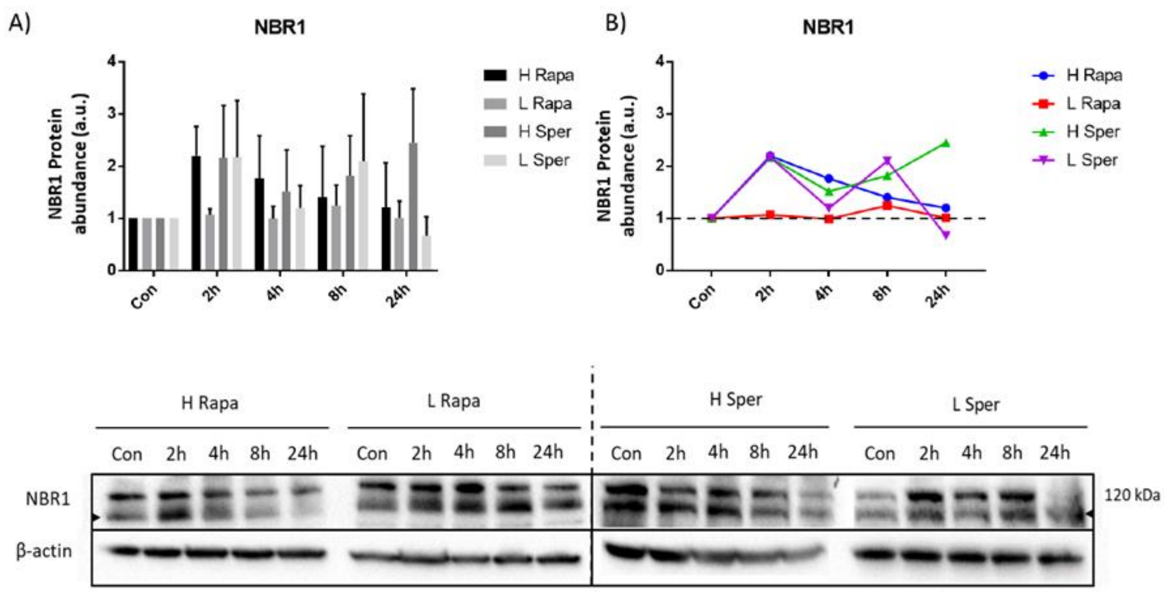

Figure S3: Western blot analysis of relative NBR1 abundance levels over 24 hours. Representative immunoblots are shown, indicating the relative abundance levels of NBR1 at 2, 4, 8 and 24 hours post-treatment using 1 µM Rapamycin (H Rapa), 10 nM Rapamycin (L Rapa), 20 µM Spermidine (H Sper) and 5 µM Spermidine (L Sper). **A)** Bar graphs and **B)** Line graphs are used to represent the data over time. N=3.

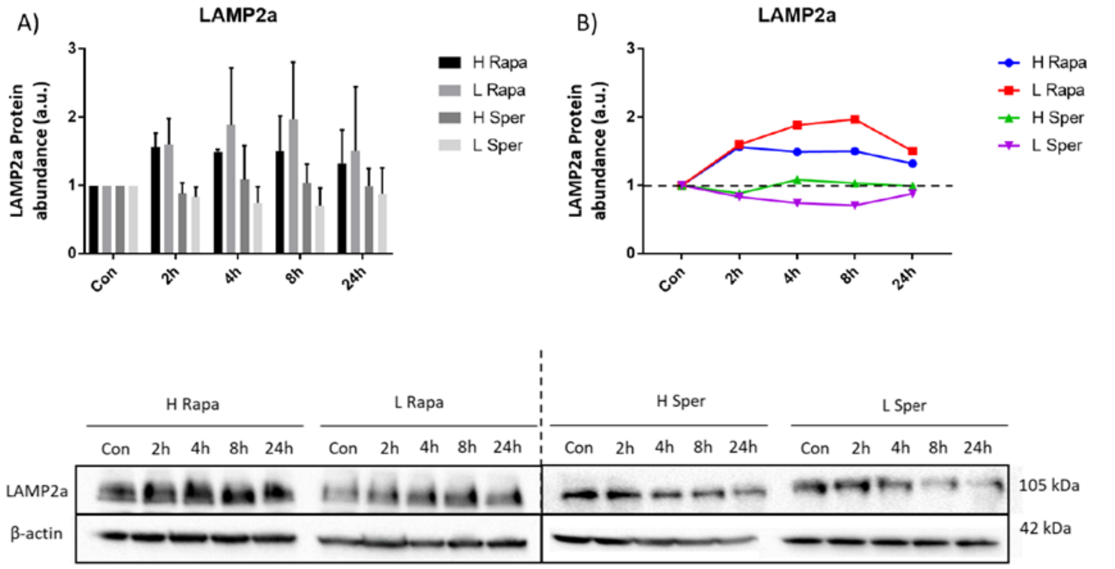

Figure S4: Western blot analysis of relative LAMP2a abundance levels over 24 hours. Representative immunoblots are shown, indicating the relative abundance levels of LAMP2a at 2, 4, 8 and 24 hours post-treatment using 1 µM Rapamycin (H Rapa), 10 nM Rapamycin (L Rapa), 20 µM Spermidine (H Sper) and 5 µM Spermidine (L Sper). **A)** Bar graphs and **B)** Line graphs are used to represent the data over time. N=3.

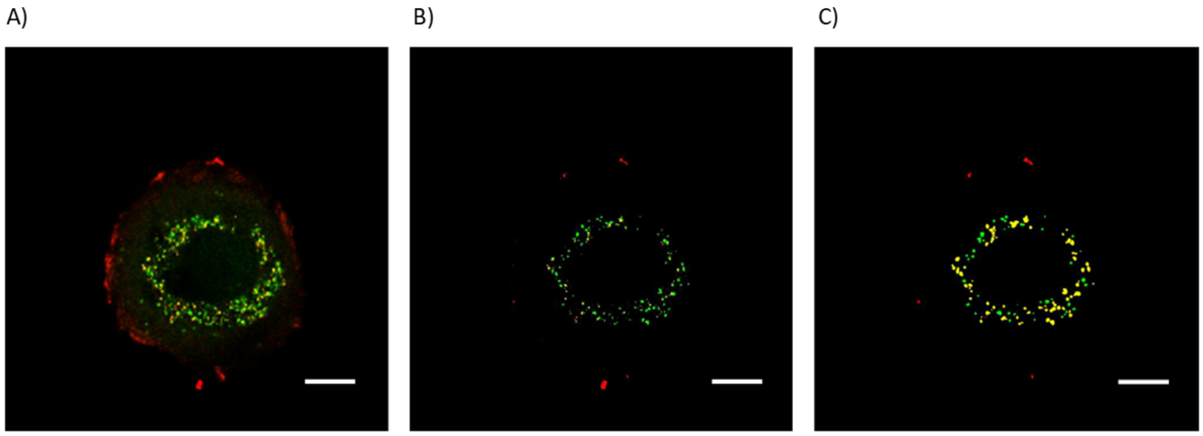

Figure S5: Representative micrographs demonstrating image processing steps. Green = GFP-LC3, red= receptor (p62), yellow= co-localised puncta. **A)** Raw image **B)** Image after adjustment **C)** Image after processing.

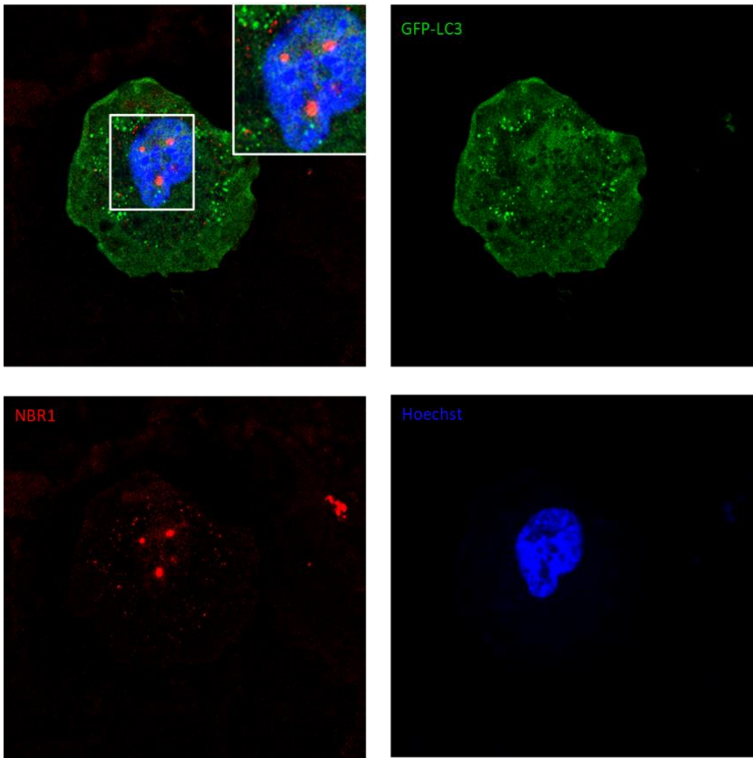

Figure S6: Representative micrograph of NBR1 positive nuclear aggregates.
